# Case report: a fatal combination of hemophagocytic lymphohistiocytosis with extensive pulmonary microvascular damage in COVID-19 pneumonia

**DOI:** 10.1007/s12308-020-00423-7

**Published:** 2020-10-23

**Authors:** Jan H. von der Thüsen, Jasper van Bommel, Johan M. Kros, Robert M. Verdijk, Boaz Lopuhaä, King H. Lam, Willem A. Dik, Jelle R. Miedema

**Affiliations:** 1 Department of Pathology, Erasmus MC, Doctor Molewaterplein 40, 3015 GD Rotterdam, The Netherlands; 2 Department of Intensive Care, Erasmus MC, Rotterdam, The Netherlands; 3 Department of Medical Immunology, Laboratory Medical Immunology, Erasmus MC, Rotterdam, The Netherlands; 4 Department of Internal Medicine, Section Clinical Immunology, Erasmus MC, Rotterdam, The Netherlands; 5 Department of Pulmonology, Erasmus MC, Rotterdam, The Netherlands

**Keywords:** COVID-19, Pneumonia, Hyperinflammatory syndrome, Microvascular thrombosis, Hemophagocytic lymphohistiocytosis

## Abstract

The clinical features of COVID-19 have a considerable range from a mild illness to severe disease. Underlying pathophysiological mechanisms of the rapidly progressive, and often fatal, pulmonary disease frequently observed in COVID-19 need to be elucidated, in order to develop new treatment strategies for different disease endotypes. Fatal cases can display features of a cytokine storm, which may be related to hemophagocytic lymphohistiocytosis. Also, a spectrum of vascular changes, including microvascular damage, is known to accompany severe COVID-19. In this paper, we describe the co-occurrence of hemophagocytic lymphohistiocytosis and extensive pulmonary microvascular damage with thrombosis and its sequelae in a patient with fatal COVID-19. We believe these response patterns may be linked by common mechanisms involving hypercytokinemia and require further investigation as a fatal constellation in COVID-19, to generate appropriate treatment in patients who display these combined features.

## Introduction

The clinical features of a patient with COVID-19 range from a mild illness with slight complaints such as sore throat and headache to patients with a very severe illness who have acute hypoxemic respiratory failure and need to be admitted to an intensive care unit. Risk factors for severe and fatal disease include elevated serum levels of D-dimer, ferritin, and interleukin (IL)-6 [1]. In addition to a study demonstrating the effect of remdesivir on time to recovery in hospital, recent evidence in favor of corticosteroid treatment to modulate inflammation in hospitalized COVID-19 patients with respiratory support is mounting [2, 3]. However, the underlying pathophysiological mechanisms of the rapidly progressive, and often fatal, pulmonary disease frequently observed in COVID-19 need to be elucidated in order to develop new treatment strategies for different disease endotypes. Hypercytokinemia has been found to correlate with occurrence of hemophagocytosis, which has been observed as a secondary phenomenon to a varying degree in viral infections. Similar mechanisms are likely to be at play in severe forms of COVID-19 and have been suggested to constitute potential targets of treatment for the cytokine storm seen in this disease [1, 4–8]. Here we present a case with histopathological evidence for hemophagocytic lymphohistiocytosis (HLH) and extensive concurrent microvascular damage in fatal COVID-19 pneumonia and speculate on the co-occurrence of these serious complications of SARS-CoV-2 infection.

## Clinical history

A 66-year-old male with a history of hypertension, peripheral arterial disease, transient ischemic attacks, and polycythemia vera presented at the emergency department with fever and shortness of breath. The patient used ruxolitinib 25 mg twice daily, acetyl salicylic acid 80 mg, atorvastatin 20 mg, calcium carbonate/colecalciferol 1.25 g/400 IE, irbesartan 300 mg, and metoprolol 200 mg once daily. On examination, he had a fever of 39.1 °C with a blood pressure of 120/90, a pulse rate of 90/min, and an oxygen saturation of 96% without a need for supplemental oxygen. His body mass index (BMI) was 36. Chest X-ray demonstrated bilateral consolidations, mostly on the right side. Polymerase chain reaction (PCR) of a nasopharyngeal swab confirmed infection with severe acute respiratory syndrome coronavirus-2 (SARS-CoV-2). Laboratory results showed progressive hyperinflammation, with a high probability of having secondary hemophagocytic lymphohistiocytosis using an online HScore calculator (Hscore 180) [9]. Additionally, elevated levels of serum cytokines and chemokines were found compared to control samples, including interleukin (IL)-1 beta (8.18 pg/ml), IL-6 (171 pg/ml), IL-6R alpha (44,035.19 pg/ml), IL-7 (16.07 pg/ml), IL-8/CXCL8 (187.75 pg/ml), IL-12p70 (92.20 pg/ml), IL-18 (5443 pg/ml), tumor necrosis factor alpha (TNFα) (12.80 pg/ml), interferon gamma (IFNγ) (2.93 pg/ml),CCL2/JE/MCP-1 (5258.93 pg/ml), CCL4/MIP-1beta (431.55 pg/ml), CXCL10/IP-10/CRG-2 (1642.41 pg/ml), galectin-9 (89,895.38 pg/ml), GM-CSF (40.77 pg/ml), and soluble IL2 receptor (1866 U/ml) (please see materials and methods section for control values). The patient deteriorated quickly and was intubated in the referring hospital and transferred to the intensive care unit in our center for invasive mechanical ventilation because of hypoxic respiratory failure. Furthermore, ruxolitinib and irbesartan were discontinued. In the subsequent days, multi-organ failure developed, including persistent respiratory insufficiency, acute kidney injury, and increased need for vasopressive therapy. Pulmonary embolism and secondary infection were excluded. The patient died of refractory multi-organ failure after 8 days of supportive therapy. His family gave consent for autopsy and publication of findings.

## Materials and methods

### Cytokine values of healthy controls (*n* = 22) (median ± standard deviation)

IL-1 beta/IL-1F2, below threshold of detection; IL-1ra/IL-1F3, 306.52 ± 225.23 (pg/ml); IL-6, 1.36 ± 0.38 (pg/ml); IL-7, 5.22 ± 1.81 (pg/ml); IL-8/CXCL8, 6.60 ± 3.98 (pg/ml); IL-10, below threshold of detection; IL-12 p70, below threshold of detection; IL-18/IL-1F4, 181.36 ± 83.43 (pg/ml); IL-6R alpha, 30,010.60 ± 6822.03 (pg/ml); TNF-alpha, 2.51 ± 0.71 (pg/ml)

### Immunohistochemistry (IHC)

Immunohistochemistry was performed with an automated, validated, and accredited staining system (Ventana Benchmark ULTRA, Ventana Medical Systems, Tucson, AZ, USA) using optiview (OV) or ultraview (UV) universal DAB detection Kit (#760-700). In brief, following deparaffinization and heat-induced antigen retrieval, the tissue samples were incubated according to their optimized time with the antibody of interest (Table 1). Incubation was followed by hematoxylin II counter stain for 12 min and then a blue coloring reagent for 8 min according to the manufactures instructions (Ventana Medical Systems).

**Table 1:** Antibody information

Antibody	Concentration	Species	Company	Clone	Pretreatment in minutes	Ab incubation time at 37 °C
CD68	0.4 μg/ml	Mouse	Ventana	KP1	CC1 8’ UV	32 min
CD163	0.2 μg/ml	Mouse	Cell Marque	MRQ-26	CC1 32’ OV	32 min
MPO	4.34 μg/ml	Rabbit	Cell Marque	polyclonal	CC1 8’ OV	32 min
CD3	0.4 μg/ml	Rabbit	Ventana	2GV6	CC1 32’ OV	32 min
CD20	0.3 μg/ml	Mouse	Ventana	L26	CC1 64’ UV	32 min
Glycophorin-C	1/800	Mouse	DAKO	Ret40f	CC1 64’ UV	32 min

### Chromogenic multiplex staining (cmIHC)

Briefly after deparaffinization, CC1 (#950-124, Ventana Medical Systems) antigen retrieval was performed for 64 min at 95 °C, followed by incubation for 8 min with the Discovery inhibitor (#760-4840, Ventana Medical Systems). Primary antibody CD163 (#760-4437, Ventana Medical Systems) was incubated for 32 min at 37 °C followed by detection with Ultramap goat-anti-mouse HRP (#760-4313, Ventana Medical Systems) and visualization using purple kit for 32 min. CC2 (#950-123, Ventana Medical Systems). A stripping step at 100 °C for 8 min was performed, followed by incubation with either CD3 (#790-4341,Ventana Medical Systems), CD20 (#760-2531,Ventana Medical Systems), MPO (#760-2659,Ventana Medical Systems), or glycophorin-C (#M0820, DAKO) at 37 °C for 32 min followed by secondary antibody and Ultramap goat-anti-mouse HRP or Ultramap goat-anti-rabbit HRP (#760-4313 and #760-4315, Ventana Medical Systems) at 37 °C for 24 min, and visualization with Teal for 32/16 min (#760-247, Ventana Medical Systems) for 32 min (Table 1). A hematoxylin II (#790-2208, Ventana Medical Systems) counter stain for 8 min and subsequent staining with a blue coloring reagent (#760–2037, Ventana Medical Systems) for 4 min were performed according to the manufacturer’s instructions (Ventana Medical Systems, Tucsen, AZ, USA). Tonsil or bone marrow was used as positive control on every slide stained.

## Results

**Fig. 1:**
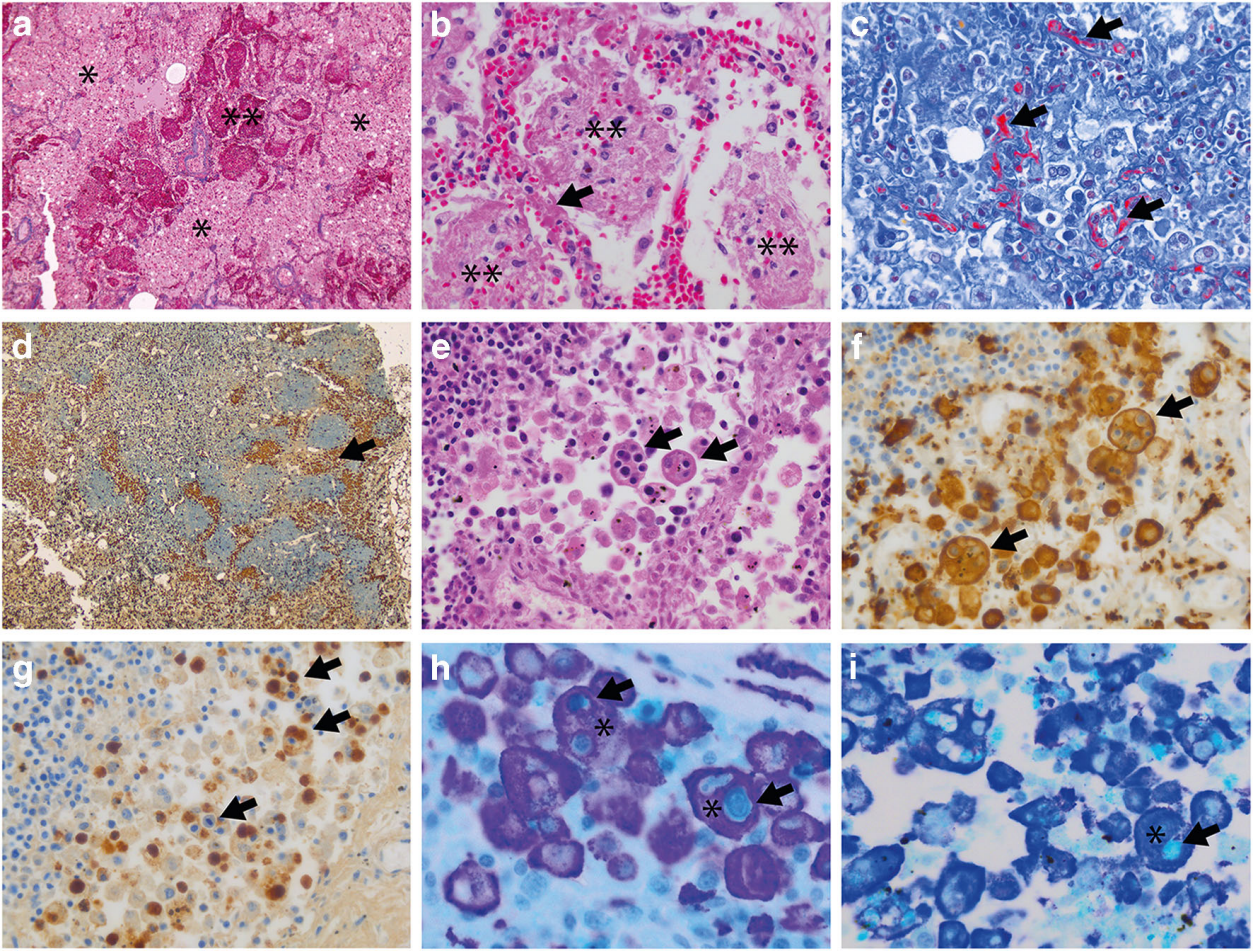
Histopathological features of microvascular damage with acute fibrinous and organizing pneumonia (AFOP) and hemophagocytic lymphohistiocytosis in a case of fatal COVID-19 pneumonia. **a** Trichrome stain showing extensive intra-alveolar edema (*) and fibrin (**). **b** Hematoxylin-eosin stain showing intra-alveolar fibrin aggregates with AFOP pattern (**) and adjacent microvascular thrombotic occlusion (arrow). **c** MSB stain of B, with in red (arrows) evident intravascular microthrombi in alveolar capillaries. **d** Peribronchial lymph node with extensive sinus histiocytosis (arrow, CD163 stain). **e** and **f** Resp. HE and CD163 stain of peribronchial lymph node demonstrating extensive hemophagocytosis (arrows). **g** MPO stain of lymph node, showing phagocytosis of granulocytes. **h** CD163 (purple) and CD3 (blue) stain, demonstrating phagocytosis of T cells. **i** CD163 (purple) and MPO (blue) stain confirming phagocytosis of granulocytes

At autopsy, bilaterally enlarged lungs were seen, with upon microscopy in the lung tissue extensive vascular changes including bilateral and diffuse edema and intra-alveolar fibrinous aggregates with an acute fibrinous and organizing pneumonia (AFOP) pattern (Fig. 1a), related to multiple associated foci of microvascular damage with hyaline thrombi (Fig. 1b and c). In a peribronchial lymph node, extensive sinus histiocytosis was seen (Fig. 1d–i), with numerous foci of hemophagocytosis, and phagocytosed cells were of the myeloid lineage, as well as T lymphocytes.

## Discussion

This case demonstrates histopathological evidence of HLH in fatal COVID-19 pneumonia with a concurrent severe microvascular damage pattern in both lungs, with capillary thrombosis and extensive intra-alveolar fibrin exudation with an AFOP pattern. This type of microvascular damage is a distinguishing feature of COVID-19 pneumonia [10], and the phenomena could be linked by a direct endothelial activation effect of multiple cytokines, including IL-6, with ensuing capillary permeability and thrombogenicity, as well as systemic hypercoagulability [4, 11, 12]. A sudden demise in COVID-19 may be related to a cytokine storm syndrome as suggested by Mehta and colleagues [10], which can be an important component of ARDS, multiple organ failure, and eventual death in SARS-CoV-2, SARS-CoV, and MERS-CoV infections. This cytokine storm is reflected by the release of large amounts of pro-inflammatory cytokines (including interferon (IFN)-α, IFN-γ, IL-1β, IL-6) and several chemokines by immune effector cells. Hypercytokinemia has been found to be correlated with the occurrence of hemophagocytosis of host cells by macrophages to a varying degree in relation to viral triggers including Epstein-Barr virus (EBV), cytomegalovirus (CMV), parvovirus, and influenza virus. The presentation of secondary HLH (sHLH) frequently includes fever, high ferritin concentrations, cytopenias, and splenomegaly. Diagnosis can be based on criteria without the need for histopathologic confirmation, and probability of secondary HLH (sHLH) can be assessed using an online calculator [9]. Recently, sHLH was found uncommon in severe COVID-19 using the calculator due to lack of some key HLH features, indicating that the HLH score has limited application in severe COVID-19 pneumonia [13]. Treatment of HLH firstly requires supportive measures to treat organ damage and secondly the elimination or specific treatment of the causative factor, which in case of SARS-CoV2 infection is currently not possible. Thirdly, suppression of the cytokine storm by immunosuppressive and cytotoxic drugs is advocated in HLH. In addition to the beneficial effects of corticosteroids, targeted immune suppression to improve mortality rates in subgroups of COVID-19 has been hypothesized [10], and multiple clinical trials with inhibition of IL-1 (e.g., anakinra) and IL-6 (e.g. tocilizumab) or Janus-kinase (JAK) pathways are currently including COVID-19 patients or revealing preliminary reports [1, 4–8, 14, 15]. While the current histopathological demonstration of HLH associated with SARS CoV2 infection further underscores the principle of using immunosuppression in a subgroup of critically ill COVID-19 patients during a second phase with signs of a cytokine storm, suppressing inflammation should always be carefully weighted up against harmfully depressing indispensable anti-viral immunity.
